# Unresolved mechanisms: a hypothesized spatial regulation of myosin light chain phosphorylation within the walls of resistance arteries

**DOI:** 10.3389/fphys.2026.1816892

**Published:** 2026-05-11

**Authors:** Zhe Sun, Michael A. Hill

**Affiliations:** 1 Dalton Cardiovascular Research Center, Columbia, MO, United States; 2 Department of Medical Pharmacology and Physiology, University of Missouri, Columbia, MO, United States

**Keywords:** mechanotransduction, myosin light chain phosphorylation, pressure myography, small resistance arteries, transmural gradient, vascular smooth muscle

## Abstract

Myosin light chain (MLC) phosphorylation is a fundamental determinant of vascular smooth muscle contraction. While its biochemical regulation has been well-studied, the spatial control of MLC phosphorylation within the vessel wall is less well understood. We recently identified a transmural gradient of phosphorylated MLC (pMLC) across the wall of rat superior cerebellar arteries, with levels peaking in outer-layer (i.e. adventitially directed) vascular smooth muscle cells (VSMCs). This finding suggests that the maintenance of vascular tone may involve spatially heterogeneous contractile signaling at the level of pMLC. We hypothesize that wall tension and consequent mechanotransduction processes to be the primary drivers of this pMLC gradient, potentially through pathways involving adhesion receptors (integrins, N-cadherin), mechanosensitive ion channels, and force-sensitive G protein-coupled receptors in outer-layer VSMCs. In addition, diffusion-limited gradients of endothelium-derived relaxing factors, including nitric oxide and prostacyclin, may establish a counter-gradient of relaxation, strongest at the inner vessel wall. The convergence of these spatially opposing signals—outer-layer mechanotransduction and inner-layer chemical relaxation—conceivably fine-tunes wall tension and stabilizes vascular tone. The proposed hypothesis is generated from evidence supporting this model of spatially regulated MLC phosphorylation as a fundamental mechanism for tension homeostasis in resistance arteries.

## Introduction

Vascular tone is fundamentally controlled by the level of phosphorylation of the myosin regulatory light chains (MLC) in vascular smooth muscle cells (VSMCs), a central event in contractile force generation (Barron et al., 1979; Kamm and Stull, 1985). Acute vasoconstriction of small arteries is typically initiated by Ca²^+^-dependent MLC phosphorylation, which in turn activates the actin-myosin contractile apparatus to induce VSM shortening and constriction. The pathways responsible for Ca²^+^ mobilization in VSMCs have been the subject of extensive investigation (Knot and Nelson, 1998; Wu et al., 1998; Jaggar et al., 2000; Hill et al., 2001). In addition, subsequent remodeling of the actin cytoskeleton has been demonstrated to be an important contributor to maintenance of VSMC contraction and is likely to be mediated by mechanisms distinct from Ca^2+^ mobilization (Cipolla et al., 2002; Flavahan et al., 2005; Kim et al., 2008; Hong et al., 2016). In comparison to the acute phase of constriction, the mechanisms by which small arteries maintain vascular tone have received less attention, even though sustained vascular contraction is a common feature in many chronic cardiovascular diseases, such as hypertension, stroke, and diabetes mellitus. Several mechanisms have been proposed to contribute to this sustained vasoconstriction including re-elongation and positioning of VSMCs in the vessel wall (Martinez-Lemus et al., 2004), and cross-linking and remodeling of extracellular matrix components (Bakker et al., 2005; Martinez-Lemus et al., 2009). Importantly, a continuum of remodeling processes of the vessel wall has been proposed to underlie the maintained vasoconstriction of small resistance arteries over a variety of time frames (i.e. from hrs. to weeks and longer) (Martinez-Lemus et al., 2009).

Arterial VSMCs adapt to changes in their surrounding mechanical environment initiating contractile and remodeling responses, as appropriate. The mechanical cues are detected by specialized mechanoreceptors which subsequently activate multiple mechanotransduction pathways that subsequently coordinate the exact cellular response (Hill and Meininger, 2012). In a recent study we reported a transmural gradient distribution of pMLC across the wall of rat superior cerebellar arteries (SCA) studied ex vivo, which peaks in the outer layer VSMCs, and diminishes towards the inner layer VSMCs (Sun et al., 2023). This finding highlights a previously understudied aspect of vascular biology, as the possible spatial regulation of pMLC is seldom considered or discussed. Intriguingly, a similar gradient—from high epicardial to low endocardial pMLC—has been documented in the heart, suggesting this may be a broader pattern in mechanically-active cardiovascular organs (Davis et al., 2001). The finding that serum induced sustained vasoconstriction of rat SCA is associated with lower pMLC levels (Sun et al., 2023) further suggests that a remodeling of the actin-myosin contractile apparatus can occur shortly (i.e. within 10 mins) after acute vasoconstriction. We propose that wall tension and the consequent activation of mechanotransduction processes in VSMCs are primary drivers of pMLC and sustain tone in small arteries. In addition, a potential gradient in the distribution of nitric oxide (NO) and perhaps other paracrine vasodilator factors such as prostacyclin may further contribute to this gradient in pMLC across the vessel wall. In regard to mechanotransduction, several mechanosensitive pathways in VSMCs are likely activated by wall tension, including mechanosensitive ion channels, force-sensitive G protein–coupled receptors (GPCRs), and adhesion junctions that transmit forces between the extracellular matrix (ECM) and the cytoskeleton. These mechanisms are discussed in more detail in the following sections.

## Mechanical considerations during sustained arterial vasoconstriction

**Figure 1:**
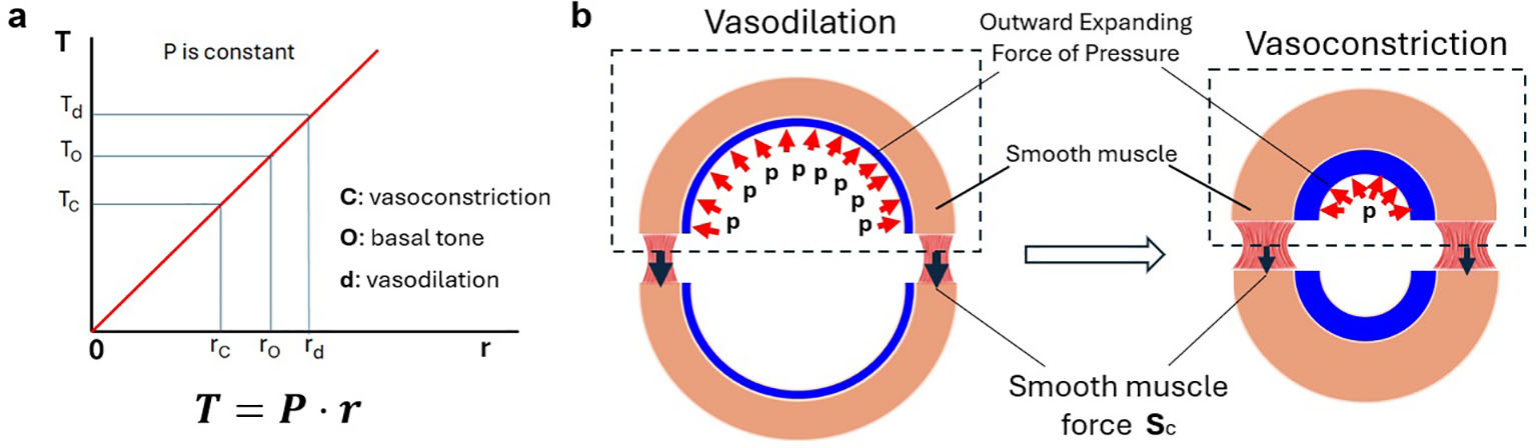
**(a)** according to LaPlace equation, vessel wall tension (T) is directly proportional to the vessel radius (r) at a constant intraluminal pressure (P). **(b)** schematic illustrating the mechanical balance in a pressurized artery wall, comparing states of vasodilation and vasoconstriction at the same intravascular pressure.

From a mechanical perspective, stable vascular tone is achieved when the contractile force generated by vessel wall VSMC is in equilibrium with the wall tension imposed by the luminal pressure, as described by a simplified Laplace equation (T = radius × pressure) (**Figure 1a**). Particularly, in studies of physics and engineering, the equation is commonly written as *Wall tension (T)=Radius × Pressure/Wall thickness*, while the Laplace equation used here assumes an infinitely thin vessel wall and estimates the total force at the cross section as shown in **Figures 1b, c**, but not wall stress. This principle dictates that under constant intraluminal pressure, wall tension is lower in constricted vessels and higher in those that have dilated (**Figures 1b, c**). As a result, the VSMC contractile force needed to maintain this steady state should be correspondingly smaller during vasoconstriction and larger during vasodilation (**Figure 1**). Consistent with this prediction, our study found that, compared to a basal level of myogenic (i.e. pressure-induced) tone, MLC phosphorylation is lower during the maintenance of serum-induced vasoconstriction, suggesting a decrease of VSMC contractile force in the vessel wall, corresponding to the lowered wall tension (Sun et al., 2023). Conversely, vasodilation that resulted from a 10 minute exposure to the myosin light chain kinase (MLCK) inhibitor, ML-7, was accompanied by an apparent increase in MLC phosphorylation, corresponding to the higher wall tension (Sun et al., 2023). As MLCK is inhibited by ML-7 in the latter condition, changes in VSMC MLC phosphorylation are likely mediated by additional kinases, such as Rho-associated coiled-coil kinase (ROCK) (Amano et al., 1996), p90 ribosomal S6 kinase 2 (RSK2) (Artamonov et al., 2018), and Zipper interacting protein kinase (ZIPK, also known as DAPK3) (Niiro and Ikebe, 2001). These findings suggest that steady-state vascular tone is maintained by the dynamic regulation of MLC phosphorylation, which fine-tunes contractile force to balance wall tension. The gradient in MLC phosphorylation across the vessel wall is a notable finding with the higher concentration of phosphorylated MLC in outer-layer VSMCs suggesting that these cells may serve as the principal force-generating component within the vessel wall. The contractile force produced by these outer-layer VSMCs likely counterbalances a substantial portion of the mechanical load arising from wall tension, in contrast, inner-layer VSMCs likely bear a smaller share of the wall tension. In agreement with this, Greensmith and Duling (using electron microscopy of cannulated arterioles) previously proposed that only the outer annulus of smooth muscle contributes to force generation in the wall of the constricted microvessel (Greensmith and Duling, 1984). This was based on the observation that myosin thick filaments aligned mostly in the tangential direction in the outer layer VSMCs of the vessel wall but were predominantly in the radial direction in the inner layer, suggesting they would not contribute to opposing maintained wall stress. To our knowledge, there remains a lack of clear evidence on how mechanical load (pressure and tension) is distributed in the vessel wall of small arteries under sustained vasoconstriction. The results discussed above are consistent with differential load-sharing across the vessel wall, i.e., the outer layer VSMCs bear a greater mechanical load than the inner layer VSMCs. This scenario further implies that mechanotransduction signaling is unevenly distributed across the vessel wall thickness with this signaling mechanism being more pronounced in outer-layer VSMCs and attenuated in inner-layer VSMCs. This imbalance in mechanosensitive signaling conceivably contributes to the observed spatial gradient in MLC phosphorylation. This also underscores the need to develop alternative vascular wall models based on vessel wall heterogeneity and represents an issue requiring attention in future investigations.

## Regulation of MLC phosphorylation in VSMC

MLC phosphorylation is dynamically regulated by the opposing activities of myosin light chain kinase (MLCK) and myosin light chain phosphatase (MLCP) (Sobieszek, 1977; Somlyo and Somlyo, 1994). MLCK is activated by the Ca²^+^-calmodulin complex, with its activity being dependent on both global cytoplasmic and localized Ca²^+^ levels. Mechanotransduction-induced localized Ca^2+^ influx/events conceivably plays an important role in the formation of a pMLC gradient in the vessel wall. In VSMCs, Ca²^+^ influx is controlled by an array of plasma membrane ion channels, including L-type voltage-gated Ca²^+^ channels (L-VGCCs) (Wu et al., 1998; Wu et al., 2001), non-selective cation TRP channels (Inoue et al., 2001; Welsh et al., 2002), and various K^+^ channels (Robertson et al., 1993; Murphy and Brayden, 1995) that modulate membrane potential. Conversely, Ca²^+^ release from the sarcoplasmic reticulum (SR) is mediated by inositol trisphosphate (IP_3_) and ryanodine receptors (RyRs) (Somlyo et al., 1985; Herrmann-Frank et al., 1991). The sequestration of Ca²^+^ back into the SR is primarily accomplished by the sarcoplasmic/endoplasmic reticulum Ca²^+^-ATPase (SERCA) pump (Jackson, 2021). Collectively, the coordinated activity of these channels and pumps precisely controls the intracellular Ca²^+^ level, thereby facilitating MLCK activation. On the opposing side, MLCP dephosphorylates pMLC, a post translational modification that inactivates myosin ATPase and promotes relaxation. A key mechanism for controlling smooth muscle contraction is the inhibitory phosphorylation of myosin light chain phosphatase (MLCP). As demonstrated in the foundational work of Kitazawa et al. (1991), activation of α_1_-adrenergic receptors, later expanded to other receptors/GPCR coupled mechanisms, triggers this pathway, inhibiting MLCP and inducing calcium sensitization. This effect is mediated primarily by the phosphorylation of the Myosin Phosphatase-Targeting Subunit 1, (MYPT1), at residues Thr-696 and Thr-853 (Human MYPT1 sequence). As detailed by Khromov et al. (2009), phosphorylated MYPT1 acts as an autoinhibitory domain that docks into the phosphatase’s active site, directly suppressing its activity. Although phosphorylation of MYPT1 fragments at both Thr-696 and Thr-853 inhibited MLCP activity *in vitro*, phosphorylation of Thr-696 appeared significantly more potent than Thr-853 both *in vitro* and in inducing contraction of β-escin-permeabilized rabbit ileum smooth muscle. Later studies further showed that phosphorylation of Thr-696 in full-length recombinant MYPT1 to be the primary inhibitory mechanism for MLCP, whereas Thr-853 phosphorylation may play an indirect and complementary inhibitory role (Khasnis et al., 2014). Furthermore, in a knock-in mouse model, the MYPT1-T853A mutation showed no apparent effect on tracheal contraction induced by carbachol treatment (Gao et al., 2017). In contrast, reduced Thr-696 phosphorylation led to decreased tone in the basilar artery isolated from aged MYPT1-T696A/+ heterozygous knock-in mice (Lubomirov et al., 2017). These authors further showed that the MYPT1-T696A mutation significantly attenuates the L-NAME-induced increase in basal tone of the basilar artery and cerebral arteries from the circle of Willis in young heterozygous knock-in mice (Lubomirov et al., 2018). The phosphorylation of Thr-853 is predominantly mediated by Rho-associated coiled-coil containing protein kinase (ROCK) (Kawano et al., 1999; Velasco et al., 2002), while phosphorylation of Thr-696 can be stimulated by multiple kinases including ROCK (Feng et al., 1999; Kawano et al., 1999; Johnson et al., 2009), integrin-linked kinase (ILK) (Kiss et al., 2002), ZIPK (Borman et al., 2002) and p21-activated kinase (PAK1) (Chu et al., 2013). In a parallel inhibitory pathway, the C-kinase-activated Protein Phosphatase-1 Inhibitor of 17 kDa (CPI-17), when phosphorylated at Thr-38, binds to the PP1c catalytic subunit of MLCP and blocks its interaction with the substrate (Etter et al., 2001; Hayashi et al., 2001; Filter et al., 2017). In summary, the regulation of smooth muscle contractility represents a balance between Ca²^+^-dependent MLCK activation and Ca²^+^-sensitization via MLCP inhibition. The inhibition of MLCP is driven primarily by the phosphorylation of MYPT1 at Thr-696, which serves as the dominant molecular switch for suppressing phosphatase activity and maintaining vasoconstriction. How these biochemical mechanisms exactly relate to spatial differences in vascular wall pMLC remain to be determined.

## VSMC adhesion receptors mediated mechanotransduction

Mechanical pulling forces applied through plasma membrane integrins and N-cadherins have been shown to induce a VSMC contractile response that has been termed a ‘micro-myogenic event’ (Sun et al., 2008; Sun et al., 2017). This contractile response to a pulling/stretching force is potentially associated with remodeling of integrin-focal adhesions or N-cadherin junctions on the cell surface (Hill and Meininger, 2012), a process that leads to spatially confined changes in phosphorylation of MLC — that is, integrin- and N-cadherin-mediated mechanotransduction. In this context, integrin signaling—which involves the Sarcoma tyrosine kinase (Src) and focal adhesion proteins such as FAK, paxillin, and vinculin—has been shown to activate L-type VGCCs (Wu et al., 1998; Wu et al., 2001) thus enhancing Ca^2+^ influx. The mechanism underlying activation involves phosphorylation of the channel’s α1C subunit at C-terminal residues Ser-1901 and Tyr-2122 (Gui et al., 2006). It has also been found that the Cav1.2 channel co-immunoprecipitated with integrin α5 and β1 in rat arteriolar VSMC and human embryonic kidney cells (HEK293-T), which depend on cell adhesion to fibronectin (FN) and require phosphorylation of Ser-1901 and Tyr-2122 (Chao et al., 2011). Collectively, this provides evidence for a mechanotransduction pathway where mechanical stress-induced integrin signaling promotes localized Ca²^+^ influx, likely in the region of focal adhesions.

**Figure 2:**
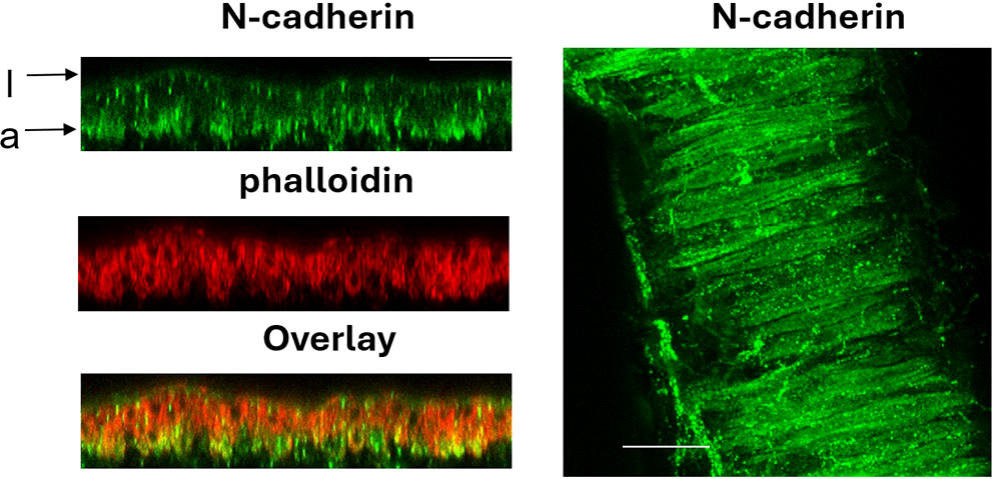
Clustering of N-cadherin to the outer layer vascular smooth muscle cell (VSMC) of pressurized rat superior cerebellar artery (SCA). Longitudinal-section view (left) and the front view of the outer layer VSMC (right) of immunofluorescent labeling of N-cadherin in the vessel wall. I: intraluminal side; a: adventitial side. Isolated rat SCA was cannulated and allowed to develop myogenic tone at 50 mmHg. Intraluminal pressure was then raised to 70 mmHg and then to 90 mmHg. After the vessel developed myogenic response at 90 mmHg and vessel diameter stabilized for 10 minutes, the SCA were fixed with 4% paraformaldehyde under pressure. Fixed SCA was immuno-labeled for N-cadherin and imaged using confocal microscopy as previously described. Scale bar=30 μm. (Z. Sun and M. Hill, unpublished data).

Integrins and N-cadherins are the principal cell-ECM and cell-cell adhesion molecules expressed in VSMCs (Davis and Hill, 1999; Jackson et al., 2010; Sun et al., 2017). In addition to modulation of VGCCs, considerable evidence suggests that integrin engagement and subsequent mechanotransduction events can activate Rho signaling (Hocking et al., 2000; Riveline et al., 2001; Matthews et al., 2006), primarily through the activation of guanine nucleotide exchange factors (GEFs) such as LARG and GEF-H1 (Marjoram et al., 2014). Similarly, N-cadherin, a major cell-cell adhesion molecule in VSMCs (Uglow et al., 2003; Sun et al., 2017), also promotes RhoA activation upon N-cadherin clustering and formation of nascent cell-cell contacts. Mechanistically, this occurs through the recruitment of RhoA-associated p120 catenin to the juxtamembrane domain of N-cadherin. RhoA is then released and can subsequently bind and activate proteins including ROCK and mDia (Mammalian Diaphanous-related formin) (Anastasiadis et al., 2000; Charrasse et al., 2002; Blindt et al., 2004). In this regard, we have observed that N-cadherin clusters in the outer layer VSMCs of vessel wall, supporting a potential role of N-cadherin-mediated mechanotransduction that conceivably drives pMLC in the outer layer VSMCs (**Figure 2**).

**Figure 3:**
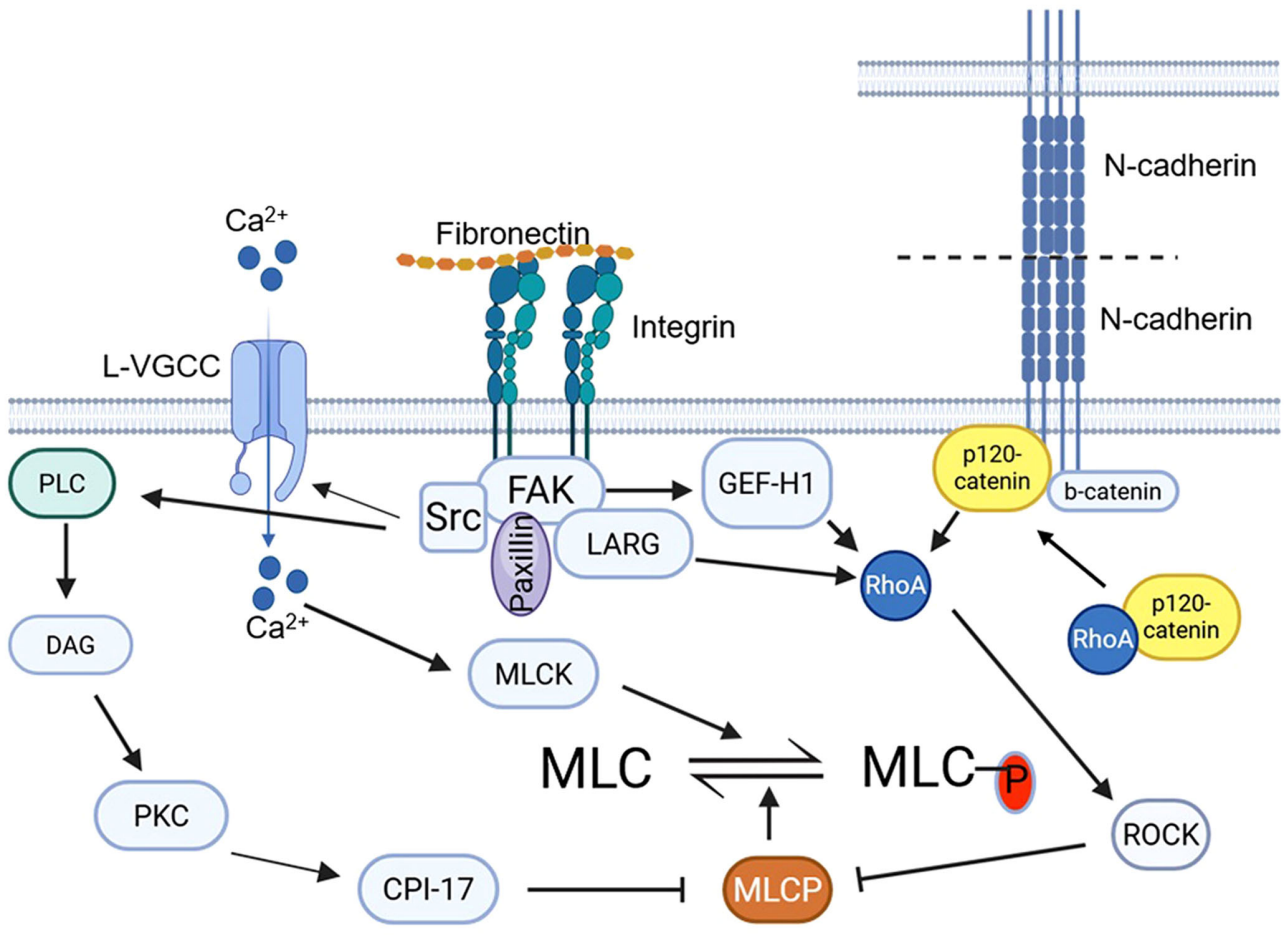
Integrin- and N-cadherin-mediated mechanotransduction signaling promotes MLC phosphorylation by regulating the balance between MLCK and MLCP. Integrin-mediated mechanotransduction and focal adhesion signaling activates Ca^2+^ influx through L-VGCC and RhoA and PKC signaling. This, in turn, stimulates MLC kinase (MLCK) and inhibits MLC phosphatase (MLCP). Similarly, N-cadherin clustering activates RhoA, leading to the inhibition of MLCP. CPI-17, C-kinase-potentiated protein phosphatase-1 inhibitor, 17 kDa; DAG −diacylglycerol; FAK, focal adhesion kinase; GEF-H1, Guanine nucleotide exchange factor H1; L-VGCC, L-type voltage gated calcium channel; LARG, Leukemia-associated Rho Guanine Nucleotide Exchange Factor; MLC, myosin light chain; MLCK, myosin light chain kinase; MLCP, myosin light chain phosphatase; PLC, phospholipase C; PKC, protein kinase C; RhoA, Ras homolog family member A; ROCK, Rho-associated coiled-coil protein kinase; Src, Proto-oncogene tyrosine-protein kinase Src.

On the basis of the above, it is plausible that vessel wall tension transmitted through both integrins and N-cadherins triggers RhoA activation. This would subsequently activate ROCK, leading to MYPT1 phosphorylation, inhibition of MLCP activity, and ultimately, localized MLC phosphorylation. (**Figure 3**) In rat models, inhibition of either integrin α5β1 or N-cadherin attenuates the myogenic response in cremaster skeletal muscle arteriole (Jackson et al., 2010) and cerebral arteries (Colinas et al., 2015). At the molecular level, increased pressure in the middle cerebral artery (MCA) induces phosphorylation of focal adhesion kinase (FAK), a Src family kinase (SFK) (as determined using a non-selective SFK antibody), vinculin, and paxillin. Critically, pharmacological inhibition of α5-integrin, FAK, or SFK blocks both the myogenic response and this cascade of adhesion protein phosphorylation. This inhibition also abrogates the downstream phosphorylation of MYPT1 (T855) and myosin light chain (S19), linking adhesion signaling directly to the contractile apparatus (Colinas et al., 2015).

An additional, and perhaps parallel, integrin-mediated mechanism may lead to inhibition of MLCP via CPI-17. In platelets, integrin αIIbβ3 ligation with fibrinogen induces Src-mediated phosphorylation and activation of Phospholipase C γ2 (PLCγ2) (Suzuki-Inoue et al., 2007). Activated PLCγ2 hydrolyzes phosphatidylinositol (4,5)-bisphosphate (PIP_2_) to generate the second messengers diacylglycerol (DAG) and inositol trisphosphate (IP_3_). While IP_3_ induces Ca²^+^ release from the sarcoplasmic reticulum, DAG—along with the elevated Ca²^+^—activates protein kinase C (PKC). Activated PKC can then phosphorylate CPI-17 at Thr-38, enabling it to potently inhibit MLCP. Thus, localized integrin-mediated mechanotransduction may also activate CPI-17, providing an additional mechanism to suppress MLCP activity and promote contraction.

Collectively, these studies support a model where integrin- and N-cadherin-mediated mechanotransduction promote localized MLC phosphorylation by concurrently stimulating Ca²^+^ influx through L-type VGCCs (driving Ca^2+^-calmodulin-dependent MLCK activity) and inhibiting MLCP activity via ROCK and CPI-17. In this regard, components of the adventitia in contact with the outer layer VSMCs are also conceivably involved in the activation of the mechanotransduction signaling pathways in the VSMCs.

## Mechanotransduction-driven localized Ca²^+^ signaling in VSMC

Multiple mechanisms have been proposed to explain intraluminal pressure-induced Ca²^+^ influx through L-VGCCs in vascular smooth muscle cells (VSMCs) (Jackson, 2021). Among them, Transient Receptor Potential Canonical (TRPC) channels are robustly expressed in VSMCs and contribute to cation entry (particularly Ca²^+^ and Na^+^), which significantly influences vascular tone. Notably, TRPC6 channels can be directly activated by membrane stretch. Spassova et al. demonstrated that this mechanosensitivity is independent of PLC-DAG signaling but is inhibited by GsMTx-4, a blocker of mechanosensitive cation channels (Bae et al., 2011), suggesting that TRPC6 senses lateral lipid tension (Spassova et al., 2006). Furthermore, Welsh et al. showed TRPC6 channels to be essential for pressure-induced depolarization in cerebral artery smooth muscle (Welsh et al., 2002). By mediating Na^+^ entry and subsequent depolarization, TRPC6 triggers secondary Ca²^+^ influx through L-VGCCs, leading to myogenic vasoconstriction. Interestingly, TRPC6-knockout animals exhibit elevated baseline blood pressure and enhanced agonist-induced contractility, a compensatory mechanism mediated by the increased expression and activity of TRPC3 (Dietrich et al., 2005). Critically, both TRPC6 and TRPC3 have been localized to the outer surface of the rat cerebral artery (Welsh et al., 2002), positioning them as suitable candidates for mediating wall tension-induced Ca²^+^ influx and the subsequent MLC phosphorylation observed in the outer-layer VSMCs of the rat superior cerebellar artery (SCA) (Sun et al., 2023). Another recently well-characterized non-selective, stretch-activated channel, Piezo1, is also highly expressed in VSMCs of small arteries, including in cerebral arteries (Retailleau et al., 2015). In cultured human aortic VSMCs, activation of Piezo 1 increases cytosolic Ca²^+^ and mediates substrate rigidity sensing (Johnson et al., 2024). However, Piezo1 knockout had no significant effect on vessel myogenic response and myogenic tone; rather, its function is linked to chronic vascular remodeling, potentially mediated by activation of the tissue type transglutaminase (Retailleau et al., 2015). Collectively, these studies outline potential mechanisms mediating localized Ca²^+^ influx in pressurized arteries. Future studies are necessary to elucidate the precise role of these mechanotransduction-driven ion channels in the formation of pMLC gradient in the vessel wall. For more detailed information on mechanosensitive ion-channels and vascular mechanotransduction, the reader is referred to the recent review by Davis et al. (2023).

## Mechanical activation of G-protein coupled receptors

Mechanical stretch and membrane distortion can also lead to Ca²^+^ influx through L-type voltage-gated calcium channels (L-VGCCs) by mechanically activating G_q/11_-coupled receptors, such as the angiotensin II type 1 receptor (AT1R), α1-adrenoreceptors and purinergic receptors (Inoue et al., 2001; Mederos y Schnitzler et al., 2008; Brayden et al., 2013; Li et al., 2014; Hong et al., 2016). Upon activation, Gαq/11 binds to and activates phospholipase C β (PLCβ), which then hydrolyzes phosphatidylinositol 4,5-bisphosphate (PIP_2_) into inositol trisphosphate (IP_3_) and diacylglycerol (DAG). This can initiate two primary signaling pathways. Firstly, DAG acts as a potent activator of TRPC6 channels, leading to membrane depolarization and subsequent secondary Ca²^+^ influx through L-VGCCs (Welsh et al., 2002). Alternatively, GPCR activation can stimulate PLCγ1, generating IP_3_ that triggers localized Ca²^+^ release from the sarcoplasmic reticulum. This Ca²^+^ release activates Ca²^+^-sensitive TRPM4 channels, contributing further to membrane depolarization and L-VGCC-mediated Ca²^+^ influx (Gonzales et al., 2010; Gonzales et al., 2014). In addition, DAG, along with Ca²^+^ released by IP_3_, may activate protein kinase C (PKC). Protein kinase C (PKC) contributes to the arterial myogenic response via multiple integrated pathways. Activated PKC further potentiates TRPM4 channel sensitivity to localized increases in Ca²^+^ concentration (Earley et al., 2007) and phosphorylates CPI-17 at Thr-38, leading to inhibition of MLCP and increased contractility. PKC also directly regulates L-VGCC clusters in VSM, promoting a persistent gating mode that generates localized, sustained Ca²^+^ influx (“Ca²^+^ sparklets”) (Navedo et al., 2005; Amberg et al., 2007). This spatial regulation is facilitated by the scaffolding protein AKAP5, which recruits PKC to the plasma membrane near L-VGCC clusters (Navedo et al., 2008). Furthermore, discrete reactive oxygen species (ROS) microdomains generated by AT1R-NADPH oxidase signaling can oxidatively activate PKCα, thereby enhancing persistent L-VGCC activity and Ca²^+^ sparklet influx (Amberg et al., 2010).

Mechanical activation of GPCRs, such as AT1R and P2Y4/P2Y6 purinergic receptors, have been shown to activate the RhoA/ROCK signaling pathway. In cardiomyocytes, mechanically stimulated AT1R is phosphorylated by G-protein-coupled receptor kinase (GRK), leading to β-arrestin recruitment. β-arrestin then scaffolds p115RhoGEF to the plasma membrane, facilitating localized RhoA activation (Rakesh et al., 2010). Downstream Rho-kinase (ROCK) activation results in MYPT1 phosphorylation and inhibition of MLCP (Ma et al., 2012). In VSMCs, P2Y4/P2Y6 receptors—coupled to both Gq/11 and G12/13—also activate the RhoA/ROCK signaling pathway (Sauzeau et al., 2000; Erb and Weisman, 2012). Specifically, in rat parenchymal arterioles (PA), mechanical activation of P2Y4/P2Y6 triggers RhoA/ROCK signaling, enhancing TRPM4 sensitivity to Ca²^+^ and thereby promoting membrane depolarization (Li and Brayden, 2017). Thus, GPCR mechanotransduction converges on several key events: diacylglycerol (DAG) generation, IP3-mediated Ca²^+^ release from the sarcoplasmic reticulum, and activation of both PKC and RhoA/ROCK signaling. We hypothesize that the outer layer VSMCs share significantly more mechanical load than the inner layer VSMCs in small arteries during sustained vasoconstriction. However, it remains unknown which mechanotransduction signaling pathways promote localized MLC phosphorylation in the outer-layer VSMCs and thus contribute to the formation of the pMLC gradient—a question that warrants further investigation.

## Mechanisms that regulate cellular localization of MLCP and MLCK:

**Figure 4:**
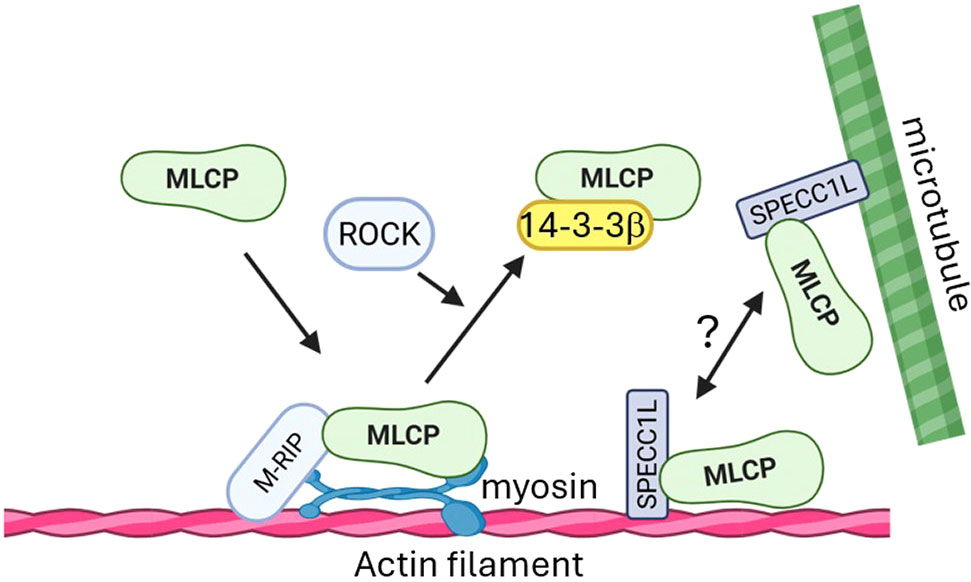
Molecular mechanism of MLCP localization. M-RIP facilitates and is required for localization of MLCP to the actin-myosin filament; while 14-3-3β could bind to MLCP and dissociate it from binding with actin-myosin filament, a process requires ROCK activity. SPECC1L is another protein that directly binds to MYPT1, localizing the MLCP complex to either actin filaments or microtubules. However, the mechanism governing this specific targeting is not well understood. 14-3-3β, Tyrosine 3-Monooxygenase/Tryptophan 5-Monooxygenase Activation Protein Beta; MLCP, myosin light chain phosphatase; M-RIP, Myosin Phosphatase Rho-Interacting Protein; ROCK, Rho-associated coiled-coil protein kinase; SPECC1L, sperm antigen with calponin homology and coiled-coil domains 1-like.

As a further mechanism by which myosin light chain phosphorylation may occur in spatially-defined regions, studies have suggested that the intracellular cellular localization of key signaling molecules may be modulated. Thromboxane-A2 has been shown to induce translocation of MYPT1 phosphorylated at phospho-Thr853 to VSMC plasma membranes in rat cerebral artery (Neppl et al., 2009). Surks et al. identified M-RIP (Myosin Phosphatase-Rho Interacting Protein) as a critical targeting regulator for the myosin light chain phosphatase. Silencing M-RIP reduced MYPT1 localization to actin stress fibers in vascular smooth muscle cells (VSMCs), leading to increased MLC phosphorylation and altered cell morphology (Surks et al., 2005). Separately, Koga and Ikebe described a novel regulatory mechanism where the 14-3–3 protein binds to MYPT1 phosphorylated at Ser472. This binding dissociates the MLCP holoenzyme from myosin II, preventing its localization to stress fibers and thereby inhibiting its activity (Takizawa et al., 2002; Koga and Ikebe, 2008). More recently, Mehta et al. identified SPECC1L (Sperm antigen with calponin homology (CH) and coiled-coil domains 1 L) as a key adaptor protein that binds both MYPT1 and the PP1β catalytic subunit. By interacting with both microtubules and actin filaments, SPECC1L serves as a “missing link” that conceivably regulates the distribution of MLCP between these cytoskeletal networks (Mehta et al., 2023). Whether these regulatory proteins contribute to the specific inhibition of MLCP in the outer-layer VSMCs of resistance arteries and whether their function is modulated by mechanical wall tension remain compelling questions for future investigation (**Figure 4**).

The short isoform of myosin light chain kinase (S-MLCK) is the predominant form expressed in smooth muscle cells. Recent work by Khapchaev demonstrated that S-MLCK can be phosphorylated *in vitro* by p44-MAPK and p38-MAPK; however, the relevant kinases responsible for its phosphorylation *in vivo* remain uncertain (Khapchaev et al., 2021). Investigations into the functional role of these sites revealed that a phospho-mimetic mutant (S25D/T56D) exhibits a diffuse distribution within cultured A10 vascular smooth muscle cells, with some concentration at the cell periphery. In contrast, a mutant (S25A/T56A) lacking the ability to be phosphorylated showed significant colocalization with the actin cytoskeleton, in addition to its peripheral localization. Neither mutation affected MLCK kinase activity, suggesting that phosphorylation at S25/T56 serves as a regulatory mechanism for S-MLCK subcellular localization. Interestingly, this phosphorylation may not be the primary driver for recruiting S-MLCK to the cell periphery, a phenomenon also observed in fibroblasts (Totsukawa et al., 2000). This peripheral concentration implies a focal adhesion-mediated recruitment mechanism and could, in turn, facilitate the localized MLC phosphorylation in the outer layer VSMCs of small arteries.

## Ca²^+^-independent direct MLC phosphorylation

Our recent work suggested a paradoxical increase in MLC phosphorylation during sustained ML-7 (15 μM)-induced dilation of rat SCA in the presence of serum (Sun et al., 2023). Since ML-7 selectively inhibits MLCK activity, this may suggest a functionally relevant, MLCK-independent pathway for MLC phosphorylation in small arteries. This possibility is supported by earlier studies from our group showing that residual pMLC levels (approximately 9%) persist in cannulated rat cremaster muscle arterioles following incubation in 0Ca^2+^/2mM EGTA (Zou et al., 1995). Such pathways could be critical for maintaining basal vascular tone and mediating localized contractile responses. The two most widely studied kinases that phosphorylate MLC at Thr18/Ser19 are ZIP kinase (ZIPK) and Rho-associated coiled-coil kinase (ROCK). ZIPK localizes to stress fibers in cultured fibroblasts (Komatsu and Ikebe, 2004) and can induce MLC phosphorylation *in vitro* and strong contraction in permeabilized arteries, even in the absence of Ca²^+^ (Niiro and Ikebe, 2001). Its physiological relevance is supported by findings that inhibiting ZIPK (also known as DAPK3) attenuates serotonin-induced vasoconstriction and pressure-induced myogenic tone in cerebral arteries (Turner et al., 2023). Furthermore, both ROCK and ZIPK, but not MLCK, are responsible for serum-induced MLC phosphorylation in cultured vascular smooth muscle cells (Deng et al., 2019). The relationship between these kinases is complex; ZIPK can be activated directly by ROCK1 through phosphorylation (Hagerty et al., 2007) and also functions downstream of TGF-β signaling during myofibroblast differentiation (Choo et al., 2024).

Beyond its canonical role of phosphorylating MYPT1 to inhibit myosin light chain phosphatase, ROCK can also directly phosphorylate MLC. This was first demonstrated *in vitro* (Amano et al., 1996) and has since been shown to induce MLC mono- and diphosphorylation in various cell types, including fibroblasts (Totsukawa et al., 2000; Newell-Litwa et al., 2015), smooth muscle (Deng et al., 2019), and endothelial cells (Kazakova et al., 2020). The subcellular localization of ROCK can be regulated by integrin-mediated focal adhesion. In CHO cells, both ROCK1 and ROCK2 concentrate in stress fiber bundles at the cell edge (Newell-Litwa et al., 2015), while in MCF7 cells, ROCKII is targeted to the plasma membrane upon cell attachment to collagen or fibronectin (Kher and Worthylake, 2011). This membrane localization of ROCKII requires both its Rho-binding domain (RBD) and pleckstrin homology (PH) domain. Collectively, these findings indicate that ROCK can directly regulate MLC phosphorylation, and that its activity and localization can be controlled by integrin-mediated focal adhesion signaling. The regulation of both ZIPK and ROCK localization within the small artery vessel wall is unclear; however, they represent a potential mechanism for inducing localized MLC phosphorylation and generating an MLC phosphorylation gradient.

In a more recent study, a novel mechanism of MLC phosphorylation by p90 ribosomal S6 kinase 2 (RSK2) has been identified (Artamonov et al., 2018). Artamonov et al. reported that RSK2 directly phosphorylates MLC at Ser19 *in vitro*, whereas mesenteric arteries from RSK2−knockout mice displayed significantly reduced myogenic tone and decreased MLC phosphorylation. Furthermore, RSK2−knockout mice exhibited significantly lower systolic blood pressure without apparent changes in cardiac function, further supporting a critical role for RSK2 in vascular function *in vivo* (Artamonov et al., 2018). Whether RSK2 plays a significant role in the formation of MLC phosphorylation gradient remains to be explored.

## Spatially graded relaxation via NO-cGMP-PKG signaling

Nitric oxide (NO) is a potent inhibitor of MLC phosphorylation in VSMCs, primarily acting through the NO-cGMP-PKG signaling axis. This pathway induces relaxation via multiple mechanisms: attenuating calcium influx, inhibiting Rho-associated kinase (ROCK), and activating myosin light chain phosphatase (MLCP) (Lincoln et al., 2001). The cGMP-dependent reduction in calcium influx is achieved through PKG-mediated phosphorylation of voltage-dependent L-type calcium channels, specifically the Cav1.2 α1c and β2 subunits (Yang et al., 2007), and by suppressing TRPC6 channel currents through PKG phosphorylation of TRPC6 at residues T70 and S322 (Koitabashi et al., 2010). Furthermore, cGMP signaling inhibits calcium release from intracellular stores; 8-Br-cGMP, a cGMP analog, has been shown to promote PKG phosphorylation of the IP3 receptor-associated protein (IRAG), thereby inhibiting IP3 receptor activity (Geiselhoringer et al., 2004). On the other hand, NO activates large-conductance, Ca^2+^-sensitive K^+^ channels (BKCa) via PKG-mediated direct phosphorylation at residue S1072 (Robertson et al., 1993; Fukao et al., 1999). Sodium nitroprusside was shown to further enhance this pathway by increasing the frequency of Ca^2+^ sparks released through ryanodine receptors (RyR) in the sarcoplasmic reticulum (SR), promoting localized BK_Ca_ activation and VSMC hyperpolarization (Porter et al., 1998; Perez et al., 2001). Beyond BK_Ca_, the NO-cGMP pathway also activates K_ATP_ channels, inducing hyperpolarization in rabbit mesenteric arteries (Murphy and Brayden, 1995), and has been implicated in Kv channel activation in the rat basilar artery *in vivo* (Sobey and Faraci, 1999). Collectively, these actions converge to lower cytosolic Ca^2+^ in VSMC, decreasing Ca^2+^-calmodulin mediated activation of MLCK.

**Figure 5:**
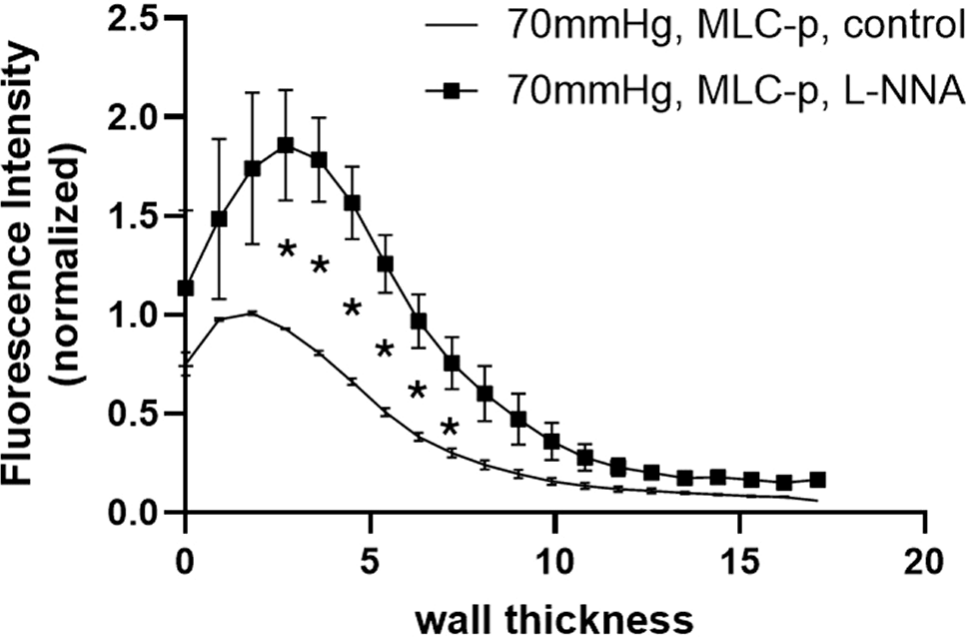
L-NNA increased level of MLC phosphorylation in the vessel wall of rat SCAs that maintain myogenic tone at 70mmHg. Wall thickness is measured by counting the stack of confocal images, from the outer surface (0 μm) to the inner surface (17 μm) of the smooth muscle layers in the vessel wall. It is estimated that one layer of VSMCs in the vessel wall is ~5 μm thick in the fixed artery. Note that the peaks of MLC phosphorylation fall within the 5 μm range, indicating they are in the outer layer VSMC. Intact SCAs were isolated, cannulated and allowed to develop myogenic tone at 50 mmHg. The lumen pressure was then increased to 70 mmHg. 10 mins after the vessel myogenic tone stabilized at 70 mmHg, SCAs were either fixed with 4% paraformaldehyde (PFA) (control group) or treated with L-NNA (10 μM) for 1 min and then fixed with 4%PFA. Vessels were immuno-labeled for phosphorylated MLC and imaged using confocal microscopy as previously described. Data was analyzed using Image J. *p<0.05, n=3 for each group. MLC-p, phosphorylated myosin regulatory light chain; L-NNA, N-nitro-L-arginine. (Z. Sun and M. Hill, unpublished data).

The NO-cGMP-PKG signaling axis relieves the inhibition of MLCP in VSMCs through multiple mechanisms. In swine carotid artery, sodium nitroprusside (SNP) increases cGMP levels, leading to a transient increase in MLCP activity that correlates with a transient decrease in CPI-17 phosphorylation (Etter et al., 2001). This reduction in CPI-17 phosphorylation is likely caused by a cGMP-mediated decrease in intracellular Ca²^+^ (Kitazawa et al., 2009). Furthermore, studies show that 8-Br-cGMP decreases MLC phosphorylation by enhancing MLCP activity. This occurs through a PKG-mediated reduction of MYPT1 phosphorylation at Thr696, a change facilitated by the concurrent phosphorylation of MYPT1 at Ser695, which sterically hinders phosphorylation at the Thr696 site (Nakamura et al., 2007). However, this working model was challenged by studies showing that the thromboxane A2 analog, U46619, induced increased phosphorylation of both Thr696 and Ser695 simultaneously in intact cerebral arteries (Neppl et al., 2009). In addition, Lubomirov et al. reported that, in femoral arteries (FA), Ser695 phosphorylation increased in response to U46619 in both control and mice with streptozotocin (STZ)-induced diabetes, while no concomitant changes in Thr696 phosphorylation were observed. On the other hand, the authors also reported that increased phosphorylation of Thr696/Thr853 partially underlies the augmented contractility of FA induced by STZ treatment, while Ser695 phosphorylation was not changed in this experimental model of diabetes (Lubomirov et al., 2019). Using rabbit femoral arterial smooth muscle strips, Kitazawa and colleagues also established that SNP-triggered dephosphorylation of CPI-17 precedes the phosphorylation of MYPT1 at Ser695. They proposed that the former initiates relaxation, while the latter maintains it (Kitazawa et al., 2009). In addition to Ser695, *in vitro* phosphorylation studies have suggested that an additional MYPT1 serine residue, Ser668, serves as the primary phosphorylation site for PKGIα (Yuen et al., 2011), and that Ser668 phosphorylation is both necessary and sufficient for PKG−mediated MLCP activation in HEK293T cells (Yuen et al., 2014). Upstream of these direct effects on MLCP, activated PKGα1 directly binds to and phosphorylates RhoA at Ser188, inactivating it and thereby preventing RhoA from activating ROCK (Kato et al., 2012). Collectively, these studies demonstrate that the NO-cGMP-PKG signaling axis leads to a decrease in MLC phosphorylation in VSMCs by both lowering cellular Ca²^+^ and alleviating the inhibition of MLCP. Consistent with this, our unpublished data have suggested a role for NO in regulating the level of pMLC in the rat SCAs maintaining myogenic tone. As shown in **Figure 5**, acute treatment with eNOS inhibitor, L-NNA, induced increased pMLC levels in cannulated rat SCAs that developed myogenic tone at 70 mmHg (**Figure 5**).

Nitric oxide (NO) has a brief half-life (0.09 to >2 seconds) in the intracellular environment, which is influenced by oxygen consumption of cells (Thomas et al., 2001). As NO diffuses through the vascular wall, it is scavenged by reactions with soluble guanylyl cyclase (sGC), protein cysteine residues (via S-nitrosylation), reactive oxygen species, and other metabolic processes. This consumption creates a steep, diffusion-limited concentration gradient across the vessel wall. The highest NO concentration is found at the interface between the endothelium and VSMC layers, while the lowest is at the outer layer VSMCs. This gradient is supported by the studies of Liu et al., who measured a 13 to 15-fold drop in NO concentration across the rat aortic wall (Liu et al., 2008). Consequently, this transmural NO gradient is predicted to cause a corresponding gradient in the inhibition of MLC phosphorylation, leading to a gradient distribution of MLC phosphorylation.

## Spatially graded relaxation via prostacyclin-cAMP-PKA signaling

Under physiological conditions within blood vessels, prostacyclin (PGI_2_) is primarily synthesized in endothelial cells by constitutively expressed cyclooxygenase-1 (COX-1) (Spisni et al., 1995; Kirkby et al., 2012). Following its synthesis, prostacyclin diffuses to the underlying vascular smooth muscle cells (VSMCs) where it activates specific Gα_s_-coupled IP receptors (Boie et al., 1994); Gα_s_ in turn triggers the adenylyl cyclase/cAMP/PKA signaling pathway. The ensuing activation of protein kinase A (PKA) downregulates MLC phosphorylation through multiple integrated mechanisms. PKA phosphorylates MLCK on specific serine residues (e.g., Ser512 and Ser525), which reduces its affinity for the Ca²^+^-calmodulin complex and thereby decreases MLCK activity (Conti and Adelstein, 1981; Ikebe and Reardon, 1990). It should be noted that these studies were performed *in vitro*, and whether PKA phosphorylates MLCK in intact smooth muscle remains to be confirmed. Similarly to PKG, *in vitro* assays have suggested that PKA could also phosphorylate MYPT1 at Ser695, thereby antagonizing ROCK-mediated inhibitory phosphorylation at Thr696 (Wooldridge et al., 2004). Furthermore, PKA phosphorylates RhoA at Ser188 and RhoGDIα at Ser174, which together result in reduced ROCK activation (Lang et al., 1996; Oishi et al., 2012). Together, these actions alleviate the inhibition of MLCP and promote relaxation.

The cAMP/PKA pathway also promotes VSMC hyperpolarization through activation of K^+^ channels. In VSMCs of rat tail and cerebral arteries, this signaling can directly activate BK_Ca_ channels, likely via PKA-mediated phosphorylation (Porter et al., 1998; Schubert et al., 1999), and can also upregulate Ca²^+^ spark frequency from RyRs, thereby increasing BK_Ca_ channel activity (Porter et al., 1998). Additionally, PKA activates K_ATP_ channels by directly phosphorylating sulfonylurea receptor 2B at S1387 (Shi et al., 2007; Shi et al., 2008). Furthermore, PKA activates K_v_1.2 channels through phosphorylation at S449 (Johnson et al., 2009). Notably, in rat cerebral artery VSMCs, this phosphorylation requires the scaffolding protein PSD-95, which binds to K_v_1.2 (Moore et al., 2014).

Owing to its spontaneous hydrolysis at physiological temperature and pH, prostacyclin has a relatively short half-life of approximately six minutes in blood (Lucas, 1986) (Lucas et al., 1986). This rapid degradation, combined with diffusion and scavenging throughout the vessel wall, could result in a concentration gradient of prostacyclin across the VSMC layers. This gradient would induce the strongest relaxation in VSMCs closest to the endothelium, with the effect diminishing toward the outer layer VSMCs.

## Conclusion

**Figure 6:**
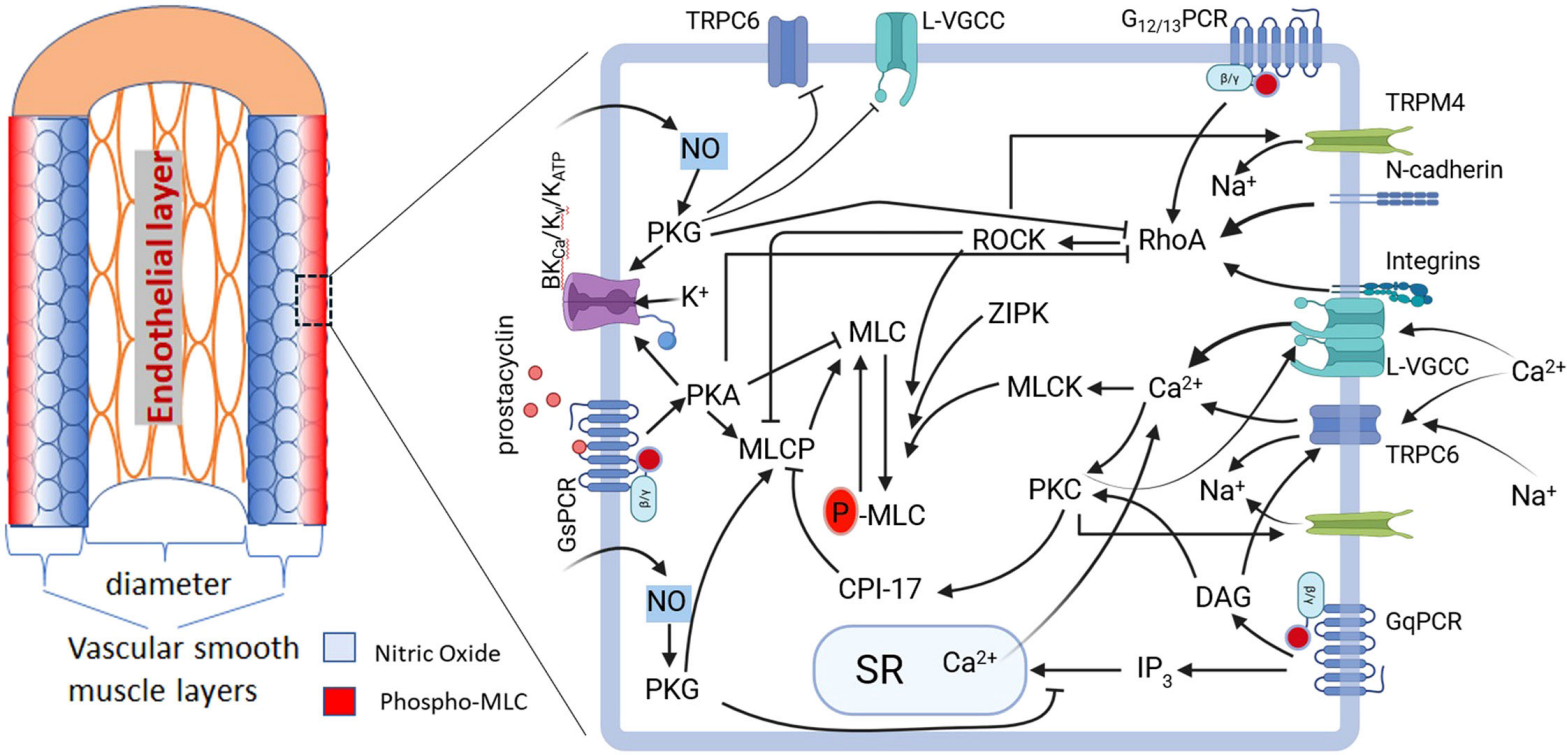
A proposed model for the establishment of MLC phosphorylation gradient in the vessel wall. The NO and prostacyclin gradient, formed via diffusion and scavenging across the VSMC layers, converges with the MLC phosphorylation gradient (red) to fine tune a mechanical equilibrium that maintains a stable vascular tone. The right panel depicts mechanotransduction pathways potentially activated by wall tension in the outer VSMCs and signaling pathways induced by NO and prostacyclin that leading to inhibition of MLC phosphorylation. CPI-17, C-kinase-potentiated protein phosphatase-1 inhibitor, 17 kDa; DAG, diacylglycerol; GPCR, G protein-coupled receptor; IP_3_, Inositol 1,4,5-trisphosphate; BK_Ca_, large conductance calcium-activated potassium channel; K_v_, voltage-gated potassium channel; K_ATP_, ATP sensitive potassium channel; L-VGCC, L-type voltage gated calcium channel; MLC, myosin light chain; MLCK, myosin light chain kinase; MLCP, myosin light chain phosphatase; NO, nitric oxide; PLC, phospholipase C; PKA, cAMP-dependent protein kinase; PKC, protein kinase C; PKG, cGMP-dependent protein kinase; RhoA, Ras homolog family member A; ROCK, Rho-associated coiled-coil protein kinase; SR, sarcoplasmic reticulum; TRPC6, transient receptor potential cation channel subfamily C member 6; TRPM4, Transient Receptor Potential Cation Channel Subfamily M Member 4; ZIPK, Zipper-Interacting Protein Kinase.

The presence of a MLC phosphorylation gradient across the vessel wall of rat SCA reveals a previously unappreciated layer of spatial regulation in the control of vascular tone. The finding implies heterogeneous force generation among VSMC layers and potential variations in mechanical load sharing. This challenges the traditional view of uniform contraction across the wall. It also suggests that the regulation of MLC phosphorylation may differ between the acute phase and the maintenance phase of vasoconstriction. Furthermore, this finding highlights a significant knowledge gap concerning the mechanisms that establish and maintain this polarized signaling pattern. While the literature on MLC phosphorylation gradient is limited (in part due to phosphorylation being typically measured in vessel wall homogenates), the pathways reviewed here suggest that localized mechanotransduction signaling, particularly at the membrane of outer layer VSMCs, could be a critical contributor in regulating MLC phosphorylation and vascular tone during the sustained phase of vasoconstriction, and may be involved in both myogenic responses and agonist-induced vasoconstrictions. This spatial regulation may be integral to a larger homeostatic system. We propose a model in which a diffusion gradient of endothelium-derived vasodilators (e.g., nitric oxide, prostacyclin) further establishes a counter-gradient of VSMC relaxation. The convergence of this relaxation gradient with the mechanotransduction-induced constriction signaling likely establishes a dynamic equilibrium, fine-tuning wall tension and stabilizing vascular tone. (**Figure 6**). Future studies should investigate whether such an MLC phosphorylation gradient exists in other vascular beds to determine if this is a specialized feature of the rat SCA or a more universal hemodynamic mechanism. Efforts should also be made to further validate the pMLC gradient on cross-sections of small arteries (e.g., mesenteric, cerebral) that under conditions of maintained vasoconstriction and fixation under pressure, using high-resolution immunohistochemistry techniques. In addition, genetically encoded tension sensors selectively expressed in smooth muscle could be developed to explore the distribution of tension across the different VSMC layers of the vessel wall. Beyond the pMLC gradient, other spatial gradients or regulatory mechanisms of contractile signaling molecules—such as Ca²^+^, RhoA, MLCP, and cGMP—remain to be explored in depth. While not emphasized at length in the manuscript, we believe these constitute important future directions.

## Data Availability

The raw data supporting the conclusions of this article will be made available by the authors, without undue reservation.
